# Author Correction: Synthesis and characterization of ZrFe_2_O_4_@SiO_2_@Ade-Pd as a novel, recyclable, green, and versatile catalyst for Buchwald–Hartwig and Suzuki–Miyaura cross-coupling reactions

**DOI:** 10.1038/s41598-024-58184-7

**Published:** 2024-04-03

**Authors:** Mohamed J. Saadh, Hussam Elddin Nabieh Khasawneh, Geovanny Genaro Reivan Ortiz, Muhammad Ahsan, Dinesh Kumar Sain, Kareem Yusuf, Mika Sillanää, Amjad Iqbal, Reza Akhavan-Sigari

**Affiliations:** 1 Faculty of Pharmacy, Middle East University, Amman, 11831 Jordan; 2 Applied Science Research Center, Applied Science Private University, Amman, Jordan; 3 Chemical Engineering Department, Al-Balqa Applied University, Salt, Jordan; 4 Laboratory of Basic Psychology, Behavioral Analysis and Programmatic Development (PAD-LAB), Catholic University of Cuenca, Cuenca, Ecuador; 5 Department of Measurements and Control Systems, Silesian University of Technology, 44-100 Gliwice, Poland; 6 Department of Chemistry, Faculty of Science, S.P. College Sirohi, Sirohi, Rajasthan 307001 India; 7 Department of Chemistry, College of Science, King Saud University, P. O. Box 2455, Riyadh, 11451 Saudi Arabia; 8 Department of Biological and Chemical Engineering, Aarhus University, Nørrebrogade 44, 8000 Aarhus, Denmark; 9 Joint Doctoral School, Silesian University of Technology, 44-100 Gliwice, Poland; 10 Department of Health Care Management and Clinical Research, Collegium Humanum Warsaw Management University, Warsaw, Poland; 11 Department of Neurosurgery, University Medical Center Tuebingen, Tuebingen, Germany

Correction to: *Scientific Reports* 10.1038/s41598-023-37680-2, published online 07 September 2023

The original version of this article contained an error in Figures 1 and 5.

**Figure 1:**
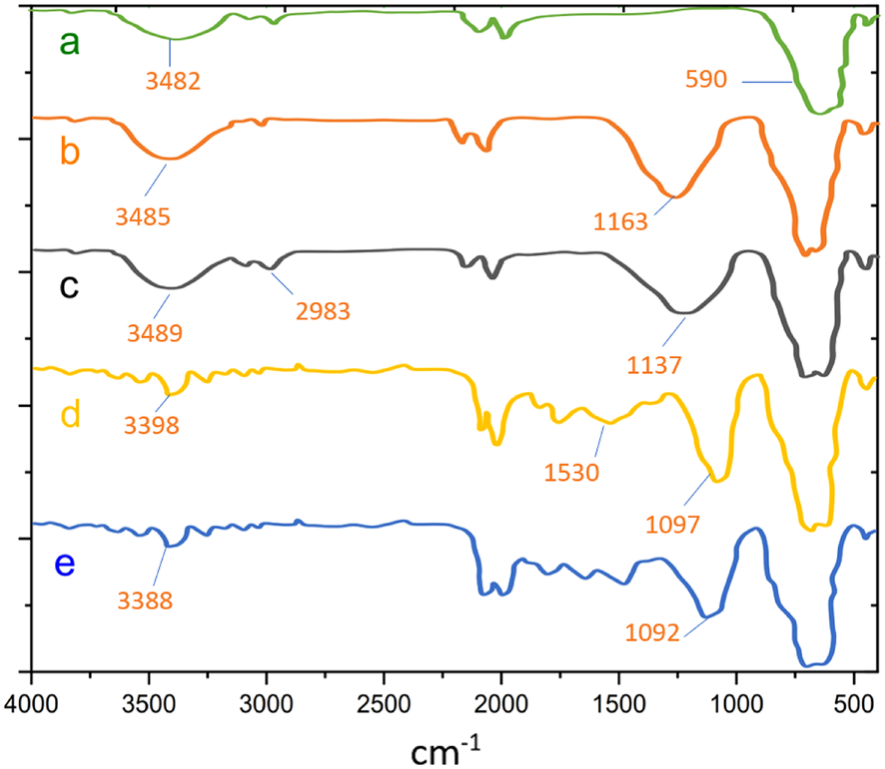
Comparative study of FTIR spectra of (**a**) ZrFe_2_O_4_, (**b**) ZrFe_2_O_4_@SiO_2_, (**c**) ZrFe_2_O_4_@SiO_2_@n-Pr (**d**) ZrFe_2_O_4_@SiO_2_@Ade, (**e**) ZrFe_2_O_4_@SiO_2_@Ade-Pd.

**Figure 5:**
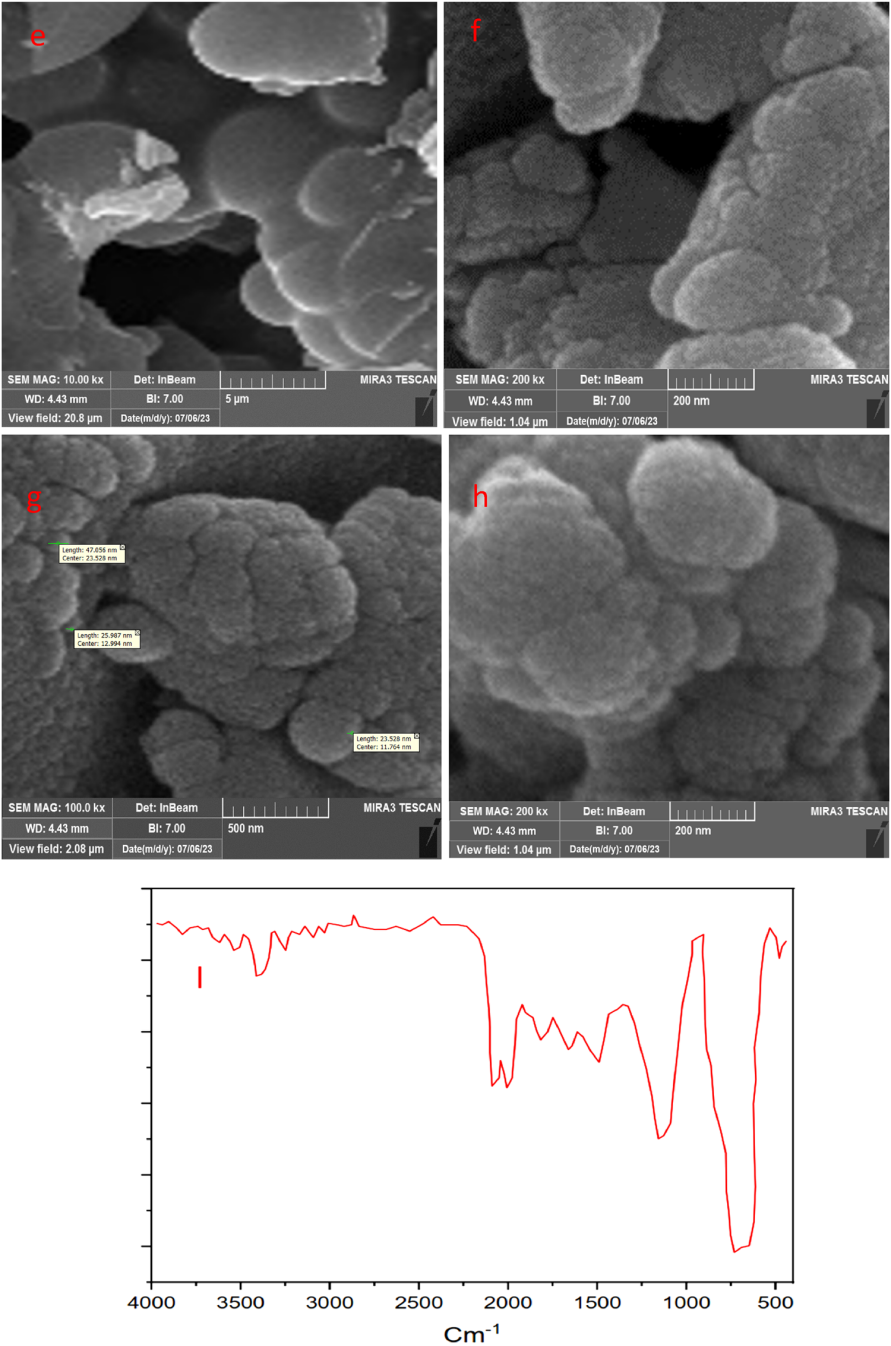
SEM (**e**–**h**) images and FT-IR spectra(I) of the recovered catalyst after five cycles.

The original Figures 1 and 5 and accompanying legends appear below.

The original article has been corrected.

